# Homologous recombination DNA repair gene *RAD51*, *XRCC2 & XRCC3* polymorphisms and breast cancer risk in South Indian women

**DOI:** 10.1371/journal.pone.0259761

**Published:** 2022-01-21

**Authors:** Taruna Rajagopal, Arun Seshachalam, Krishna Kumar Rathnam, Srikanth Talluri, Sivaramakrishnan Venkatabalasubramanian, Nageswara Rao Dunna

**Affiliations:** 1 Cancer Genomics Laboratory, Department of Biotechnology, School of Chemical and Biotechnology, SASTRA–Deemed University, Thanjavur, India; 2 Department of Medical and Paediatric Oncology, Dr.G.V.N Cancer Institute, Singarathope, Trichy, India; 3 Department of Hemato Oncology–Medical Oncology and Bone Marrow Transplantation, Meenakshi Mission Hospital & Research Centre, Madurai, India; 4 Dana Farber Cancer Institute, Boston, MA, United States of America; 5 Veterans Administration Boston Healthcare System, West Roxbury, MA, United States of America; 6 Department of Genetic Engineering, Faculty of Engineering and Technology, SRM Institute of Science and Technology, Kattankulathur Campus, Chennai, India; CNR, ITALY

## Abstract

**Background:**

Homologous recombination repair (HRR) accurately repairs the DNA double-strand breaks (DSBs) and is crucial for genome stability. Genetic polymorphisms in crucial HRR pathway genes might affect genome stability and promote tumorigenesis. Up to our knowledge, the present study is the first to investigate the impact of HRR gene polymorphisms on BC development in South Indian women. The present population-based case-control study investigated the association of polymorphisms in three key HRR genes (*XRCC2*-Arg188His, *XRCC3*-Thr241Met and *RAD51*-G135C) with BC risk.

**Materials and methods:**

Polymerase chain reaction-restriction fragment length polymorphism (PCR-RFLP) method was used for genotyping the HRR variants in 491 BC cases and 493 healthy women.

**Results:**

We observed that the *XRCC3* Met allele was significantly associated with BC risk [OR:1.27 (95% CI: 1.02–1.60); p = 0.035]. In addition, the homozygous mutant (C/C) genotype of *RAD51* G135C variant conferred 2.19 fold elevated risk of BC [OR: 2.19 (95% CI: 1.06–4.54); p = 0.034]. Stratified analysis of HRR variants and BC clinicopathological features revealed that the *XRCC3*-Thr241Met and *RAD51*-G135C variants are associated with BC progression. Combined SNP analysis revealed that the individuals with *RAD51*-C/C, *XRCC2*-Arg/Arg, and *XRCC3*-Thr/Thr genotype combination have three-fold increased BC risk.

**Conclusion:**

The present study imparts additional evidence that genetic variants in crucial HRR pathway genes might play a pivotal role in modulating BC risk in South Indian women.

## Introduction

Breast cancer (BC) is a complex polygenic disease that arises due to the synergistic effect of several genetic variations and environmental factors. Molecular epidemiological studies have suggested that approximately 80% of BC’s inherited susceptibility is due to the combined effect of several low penetrant gene variants rather than the high penetrant gene mutations [1]. Moreover, it has been observed that most of the epidemiological studies have been conducted on participants from European ancestry. Therefore, simultaneously increasing the representation of participants from other populations is highly recommended [2]. BC possesses a significant health burden in both developed and developing countries. Surprisingly, the highest BC incidence rate was observed in the Chennai (South Indian city) registry, and the BC burden was estimated to escalate up to 233 per 1000 females by 2026 in India [3]. Furthermore, the impact of candidate gene polymorphisms on BC risk has not been completely ascertained in South Indian women.

Several endogenous or exogenous factors trigger aggressive DNA damages, such as double-strand break (DSB) lesions, which are generally repaired by the DSB repair. DSB is repaired via two pathways: Homologous recombination repair (HRR) and non-homologous end joining (NHEJ) DSB repair. HRR genes function as genomic caretakers, and germline mutations in crucial HRR genes have been strongly associated with tumor predisposition [4, 5]. In HRR, the MRN (MRE11/RAD50/NBN) complex binds to the ends of DSBs. The nucleotides from the 5’ end of DSBs are excised by MRE11, thereby resulting in 3’ single-strand DNA (ssDNA) overhangs [6]. *RAD51* forms a nucleoprotein filament by binding to ssDNA and facilitates strand invasion into homologous DNA duplex using various mediator proteins such as XRCC2, XRCC3, and BRCA2. The newly synthesized DNA then dissociates to anneal with an opposite DNA strand, and ligation completes the HRR process [7, 8]. A G>C substitution (rs1801320) at position 135, in the 5’ untranslated region (UTR) of *RAD51* has been reported as a modulator of *RAD51* DNA repair capacity (DRC). Individuals with the C allele had the lowest DRC, thereby suggesting the *RAD51* G135C variant has a functional role in modulating BC susceptibility [9].

Similarly, X-ray repair cross-complementing 2 (*XRCC2*), a member of the RAD51 family of proteins, possess walker motifs A and B (which are ATP binding domains) and is a crucial protein that mediates HRR [10, 11]. Interestingly, XRCC2 functions as an enhancer of RAD51 activity, and loss of XRCC2 protein activity results in a critical delay in the initial RAD51 response to the DNA damage [12]. A non-synonymous variation (rs3218536) caused due to c.563G>A substitution in exon 3 of *XRCC2* gene results in substitution of Arg to His amino acid at codon 188. Moreover, site-directed mutagenesis of *XRCC2* revealed that non-conservative amino acid substitution at 188th amino acid position significantly affects cell’s sensitivity to DNA damage [13]. Furthermore, the *XRCC2* Arg188His variant was found to modify BC risk in women with reduced plasma folate levels [14]. Likewise, *XRCC3*, a *RAD51* paralog, controls the fidelity of HR and is essential for stabilising heteroduplex DNA in HRR. Furthermore, a mutation in X-ray repair cross-complementing 3 (*XRCC3)* generates severe chromosomal instability [15]. A common variant (rs861539) in the *XRCC3* gene is a c.722C>T substitution in exon 7, which results in Thr to Met amino acid substitution at codon 241. Additionally, individuals carrying the Met allele had increased DNA adduct levels in the lymphocyte DNA [16, 17]. Moreover, *in vitro* studies suggested that the *XRCC3*-241Met variant increased an individual’s cancer risk [18]. Besides, Song *et al*. conducted a meta-analysis and highlighted that the Thr241Met variant was significantly associated with a higher risk of radiation-induced early adverse outcomes, as well as specific detrimental effects such as mucositis and acute skin toxicity [19].

Last decade, there have been conflicting reports regarding the impact of the HRR gene polymorphisms on BC risk [20–26]. Besides, there is a paucity of information regarding the impact of HRR gene polymorphisms on South Indian women’s BC etiology. Hence, we conducted this population-based case-control study to evaluate the impact of *XRCC2*-Arg188His, *XRCC3*-Thr241Met, and *RAD51*-G135C variants with BC risk in South Indian women.

## Materials and methods

### Study subjects

The present study investigated 984 subjects, which included 491 histopathologically confirmed breast cancer cases and 493 healthy women from South India. The institutional ethics committee of Dr. G.V.N Cancer Institute (ECR/436/INST/TN/2013) and MMHRC (ECR/398/INST/TN/2013/RR-16) approved the present study. The samples were collected following the tenets of the Declaration of Helsinki and its later amendments. Written informed consent was obtained from the study participants. Peripheral blood of BC patients was collected from the medical oncology department of Dr. G.V.N Cancer Institute and Meenakshi Mission Hospital & Research Centre between June 2017 and January 2020. Blood samples of healthy (cancer-free) women, who are age and ethnicity matched to cases and without a family history of cancer, were collected during the same period. The surgically resected primary tumors of the BC patients were graded according to the Scarf-Bloom-Richardson grading system and staged based on the American Joint Committee of Cancer (AJCC) system. Clinico-pathological characteristics of the BC patients such as menopausal status, age at disease onset, hormonal receptor status, tumor grade, tumor stage, and metastasis extent were noted with the help of a medical oncologist.

### Genomic DNA extraction

Antecubital venepuncture was performed to draw 3-5ml of venous blood from the study participants in a commercially available sterile K2-EDTA coated vacutainer (BD Vacutainer®, Franklin Lakes, USA). The genomic DNA was extracted from the whole blood samples using the HiPurA SPP blood DNA isolation kit (HiMedia^TM^, Mumbai, India) following the manufacturer’s protocol. The isolated DNA was quantitatively assessed for purity using the NanoDrop 2000^TM^ spectrophotometer (Thermo Fischer Scientific, USA). All the genomic DNA samples were stored at -20°C until further analysis.

### Genotyping

PCR–RFLP (Polymerase Chain Reaction–Restriction Fragment Length Polymorphism) method was used for genotyping *XRCC2*-Arg188His, *XRCC3*-Thr241Met, and *RAD51*-G135C variants. The PCR reactions were carried out in a final volume of 25μl reaction mixture comprising 12.5μl of 2x GoTaq Green master mix (Promega, Madison, USA), 0.5μl of each primer (10μM), 100-150ng genomic DNA, and nuclease-free water. PCR reactions were carried out using T100^TM^ thermal cycler (Bio-Rad, CA, USA). The PCR amplicons were run on 1% ethidium bromide-stained agarose gel and visualized using Bio-Rad XR^+^ gel documentation system (Bio-Rad, CA, USA). The list of primers, annealing temperature, and RFLP conditions utilized is given in Table 1. The investigated variants’ genotypes were determined by performing electrophoresis of the digested PCR products in ethidium bromide-stained 4% agarose gel and visualized using a gel documentation system (Bio-Rad, CA, USA). To assess the genotyping quality, genotyping was repeated in random 10% of the samples, and the results were 100% concordant.

**Table 1:** PCR conditions, PCR primers and RFLP pattern.

Gene; SNP	PCR Primers	Annealing Temperature	Restriction enzyme	RFLP pattern
*XRCC2*;	**F**: 5’- TGTAGTCACCCATCTCTCTGC -3’	58°C	*HphI*	Arg/Arg: 290 bp
rs3218536; (G>A)	**R**: 5’- AGTTGCTGCCATGCCTTACA -3’			Arg/His: 290 bp, 148 bp & 142 bp
				His/His: 148 bp & 142 bp
*XRCC3;*	**F**: 5’- GCCTGGTGGTCATCGACTC -3’	61°C	*NcoI*	Thr/Thr: 136 bp
rs861539; (C>T)	**R**: 5’-ACAGGGCTCTGGAAGGCACTGCTCAGCT CACGCACC -3’			Thr/Met: 136 bp, 97 bp & 39 bp
				Met/Met: 97 bp & 39 bp
*RAD51;*	**F**: 5’- TGGGAACTGCAACTCATCTGG -3’	60°C	*MvaI*	G/G: 86 bp & 71 bp
rs1801320; (G>C)	**R**: 5’- GCGCTCCTCTCTCCAGCA- 3’			G/C: 157 bp, 86 bp & 71 bp
				C/C: 157 bp

F: Forward Primer; R: Reverse Primer; bp: Base pair

### Statistical analysis

With respect to the SNPs investigated, the genotypes of the controls were assessed for their agreement with Hardy-Weinberg equilibrium (HWE) using the χ2 goodness of fit test. Odds ratio (OR) and 95% confidence interval (CI) were determined by performing unconditional logistic regression analysis using SNPStats online software (https://www.snpstats.net/). Stratified analysis was carried out between the investigated SNPs and clinicopathological features of BC cases to evaluate SNPs’ role in disease progression. P-value <0.05 was considered as statistically significant. Multifactor-dimensionality reduction (MDR) analysis and interaction dendrogram was constructed using the MDR software package (MDR 3.0.2) to evaluate the impact of gene-gene interaction on BC risk. The best interaction model was selected based on the highest cross-validation consistency (CVC) and testing balance accuracy (TBA). Further, STRING software was used to visualize protein-protein interaction (https://string-db.org/).

## Results

### Characteristics of the study population

The present study comprised of 491 breast cancer cases and 493 healthy women as controls. The mean age of BC onset in the patients was 52.1±10.99 yrs. The clinicopathological features of BC cases are summarized in Table 2.

**Table 2:** Demographic and clinicopathological characteristics of the study subjects.

Characteristics	BC cases n (%)	Controls n (%)
**Age in years (mean±S.D)**	52.51±10.99	51.40±14.48
**Menopausal Status**		
Pre-menopause	148 (30.1)	157 (31.8)
Post-menopause	343 (69.9)	336 (68.2)
**Molecular Subtype**		
Luminal	292 (59.4)	
Her2 enriched	102 (20.8)	
TNBC	97 (19.8)	
**Tumor Stage**		
Early (T1+T2)	305 (62.1)	
Advanced (T3+T4)	186 (37.9)	
**Histological Grade**		
Low (GI)	81 (16.5)	
High (GII+GIII)	410 (83.5)	
**Metastasis**		
Positive	120 (24.4)	
Negative	371 (75.6)	
**Estrogen Receptor**		
Positive	278 (56.6)	
Negative	213 (43.4)	
**Progesterone Receptor**		
Positive	212 (43.2)	
Negative	279 (56.8)	
**HER2/neu**		
Positive	204 (41.5)	
Negative	287 (58.5)	

n: number; HER2: Human Epidermal Growth Factor Receptor 2; TNBC: Triple-Negative Breast Cancer.

### Hardy-Weinberg Equilibrium (HWE) test

The observed genotype frequency for the studied polymorphic loci were in accordance with Hardy-Weinberg equilibrium in the controls.

### Allele and genotype distribution of *XRCC2*-Arg188His, *XRCC3*-Thr241Met, and *RAD51*-G135C variants

#### XRCC2-Arg188His variant analysis

Genotype frequency distribution of the *XRCC2*-Arg188His variant (Arg/Arg, Arg/His and His/His genotypes) was 79.9%, 19.3% and 0.8% in controls and 76.6%, 21.6% and 1.8% in BC cases, respectively. However, no significant association was observed between the genotype and allele frequency distribution of the *XRCC2*-Arg188His variant in BC cases and controls (Table 3) (S1 File).

**Table 3:** Allele and genotype frequencies of *XRCC2*, *XRCC3* and *RAD51* polymorphisms in BC cases and controls.

Model	Genotype & Allele	BC Cases N = 491	Controls N = 493	OR (95%CI)^a^	*p -*value
***XRCC2* (Arg188His)**					
Co-dominant	Arg/Arg	376 (76.6**%**)	394 (79.9**%**)	Reference	
	Arg/His	106 (21.6**%**)	95 (19.3**%**)	1.17 (0.86–1.60)	0.324
	His/His	9 (1.8**%**)	4 (0.8**%**)	2.36 (0.72–7.72)	0.156
Dominant	Arg/Arg	376 (76.6**%**)	394 (79.9**%**)	Reference	
	Arg/His + His/His	115 (23.4**%**)	99 (20.1**%**)	1.22 (0.90–1.65)	0.200
Recessive	Arg/Arg + Arg/His	482 (98.2**%**)	489 (99.2**%**)	Reference	
	His/His	9 (1.8**%**)	4 (0.8**%**)	2.28 (0.70–7.46)	0.172
Allele	Arg	858 (87.4**%**)	883 (89.6**%**)	Reference	
	His	124 (12.6**%**)	103 (10.4**%**)	1.24 (0.94–1.64)	0.130
***XRCC3* (Thr241Met)**					
Co-dominant	Thr/Thr	310 (63.1**%**)	342 (69.4**%**)	Reference	
	Thr/Met	158 (32.2**%**)	134 (27.2**%**)	1.30 (0.99–1.72)	0.062
	Met/Met	23 (4.7**%**)	17 (3.4**%**)	1.49 (0.78–2.85)	0.223
Dominant	Thr/Thr	310 (63.1**%**)	342 (69.4**%**)	Reference	
	Thr/Met + Met/Met	181 (36.9**%**)	151 (30.6**%**)	**1.32 (1.01–1.72)**	**0.038**
Recessive	Thr/Thr + Thr/Met	468 (95.3**%**)	476 (96.6**%**)	Reference	
	Met/Met	23 (4.7**%**)	17 (3.4**%**)	1.38 (0.73–2.61)	0.330
Allele	Thr			778 (79.2**%**)	818 (83.0**%**)	Reference	
	Met	204 (20.8**%**)	168 (17.0**%**)	**1.27 (1.02–1.60)**	**0.035**
***RAD51* (G135C)**					
Co-dominant	G/G	372 (75.8**%**)	374 (75.9**%**)	Reference	
	G/C	95 (19.3**%**)	108 (21.9**%**)	0.88 (0.65–1.21)	0.438
	C/C	24 (4.9**%**)	11 (2.2**%**)	**2.19 (1.06–4.54)**	**0.034**
Dominant	G/G	372 (75.8**%**)	374 (75.9**%**)	Reference	
	G/C + C/C	119 (24.2**%**)	119 (24.1**%**)	1.01 (0.75–1.35)	0.970
Recessive	G/G + G/C	467 (95.1**%**)	482 (97.8**%**)	Reference	
	C/C	24 (4.9**%**)	11 (2.2**%**)	**2.25 (1.09–4.65)**	**0.023**
Allele	G	839 (85.4**%**)	856 (86.8**%**)	Reference	
	C	143 (14.6**%**)	130 (13.2**%**)	1.12 (0.87–1.45)	0.377

p <0.05 is considered as significant; OR odds ratio; CI, confidence interval; n: number; a–crude OR

#### XRCC3-Thr241Met variant analysis

The genotype (Thr/Thr, Thr/Met and Met/Met) frequency distribution of the *XRCC3*-Thr241Met variant was found to be 69.4%, 27.2% and 3.4% in the controls and 63.1%, 32.2% and 4.7% in BC cases respectively (Table 3). A marginal association was observed between the heterozygous genotype and BC risk, where the Thr/Met genotype conferred 1.30-fold elevated BC risk; however, the association was insignificant (p>0.05). Under the dominant model, we observed that the Thr/Met + Met/Met genotype conferred an 1.32-fold elevated risk of BC development (OR:1.32 [95% CI: 1.01–1.72]; p = 0.038). Similarly, the allele frequency distribution of the Thr and Met alleles were found to be 83% and 17% in the controls and 79.2% and 20.8% in the BC cases. Interestingly, the Met allele was significantly associated with BC risk [OR:1.27 (95% CI: 1.02–1.60); p = 0.035] in South Indian women (Table 3).

#### RAD51-G135C variant analysis

The frequency of G/G, G/C, and C/C genotypes of the *RAD51*-G135C variant in the controls were found to be 75.9%, 21.9%, and 2.2%, whereas it was observed to be 75.8%, 19.3%, and 4.9% in BC cases, respectively. We noticed that the homozygous mutant (C/C) genotype conferred 2.19-fold elevated risk of developing BC [OR: 2.19 (95% CI: 1.06–4.54); p = 0.034]. We additionally observed that the C/C genotype conferred 2.25-fold [OR: 2.25 (95% CI: 1.09–4.65); p = 0.023] elevated risk of BC under the recessive inheritance model (G/G+G/C vs. C/C) (Table 3).

### Association between HRR gene polymorphisms and BC clinicopathological characteristics

To evaluate the association between the HRR gene polymorphisms and various BC clinicopathological features, BC patients were stratified based on their genotypes and clinicopathological characteristics. Pertaining to the *XRCC3* Thr241Met variant, the heterozygous (Thr/Met) genotype reduced the risk of developing higher-grade tumors [OR: 0.58 (95% CI: 0.35–0.95); p = 0.031] (Table 4).

**Table 4:** Evaluation of *XRCC3* and *RAD51* variants with BC patients’ clinicopathological features.

CLINICAL VARIABLES	*XRCC3* Thr241Met			*RAD51* G135C		
	Thr/Thr	Thr/Met	Met/Met	G/G	G/C	C/C
**Tumor grade**						
High/Low	266/44	123/35	21/2	310/62	81/14	19/5
OR (95% CI)	Reference	**0.58 (0.35–0.95)**	1.74 (0.39–7.67)	Reference	1.16 (0.62–2.17)	0.76 (0.27–2.11)
*p*-value		0.031	0.466		0.649	0.599
**Tumor Stage**						
III+IV/II+I	116/194	61/97	9/14	136/236	40/55	10/14
OR (95% CI)	Reference	1.05 (0.71–1.56)	1.07 (0.45–2.56)	Reference	1.26 (0.80–1.99)	1.24 (0.54–2.87)
*p*-value		0.802	0.870		0.320	0.616
**HER2/neu status**						
+ve/-ve	136/174	58/100	10/13	155/217	39/56	10/14
OR (95% CI)	Reference	0.74 (0.50–1.10)	0.98 (0.42–2.31)	Reference	0.98 (0.62–1.54)	1.00 (0.43–2.31)
*p*-value		0.138	0.971		0.914	1.000
**ER Status**						
-ve/+ve	134/176	67/91	12/11	166/206	40/55	7/17
OR (95% CI)	Reference	0.97 (0.66–1.42)	1.43 (0.61–3.35)	Reference	0.90 (0.57–1.42)	0.51 (0.21–1.26)
*p*-value		0.865	0.406		0.659	0.145
**PR Status**						
-ve/+ve	173/137	90/68	16/7	216/156	51/44	12/12
OR (95% CI)	Reference	1.05 (0.71–1.54)	1.81 (0.72–4.52)	Reference	0.84 (0.53–1.32)	0.72 (0.32–1.65)
*p*-value		0.812	0.204		0.442	0.440
**Metastasis**						
+ve/-ve	76/234	38/120	6/17	85/287	25/70	10/14
OR (95% CI)	Reference	0.98 (0.62–1.52)	1.09 (0.41–2.86)	Reference	1.21 (0.72–2.02)	**2.41 (1.03–5.62)**
*p*-value		0.912	0.866		0.478	0.042
**Age at onset (years)**						
≤40 / >40	40/270	26/132	6/17	58/314	10/85	4/20
OR (95% CI)	Reference	1.33 (0.78–2.27)	2.38 (0.89–6.40)	Reference	0.64 (0.31–1.30)	1.08 (0.36–3.28)
*p*-value		0.298	0.085		0.215	0.888
**BMI**						
3+4 / 2+1	196/114	117/41	11/12	256/116	53/42	15/9
OR (95% CI)	Reference	**1.66 (1.09–2.54)**	0.53 (0.23–1.25)	Reference	**0.57 (0.36–0.91)**	0.75 (0.32–1.78)
*p*-value		0.019	0.147		0.017	0.519
**Menopausal status**						
Pre/Post	84/226	53/105	11/12	117/255	22/73	9/15
OR (95% CI)	Reference	1.36 (0.89–2.05)	**2.47 (1.05–5.80)**	Reference	0.66 (0.39–1.11)	1.31 (0.56–3.07)
*p*-value		0.148	0.039		0.116	0.539

*p-*value < 0.05 is considered as significant and are highlighted in bold; OR: Odds ratio; CI: Confidence interval; BMI: Body Mass Index: 4- Obese; 3 –Overweight; 2- Normal weight; 1 –Underweight; ER: Estrogen Receptor; PR: Progesterone Receptor; HER2: Human Epidermal Growth Factor Receptor 2.

However, in contrast, the heterozygous (Thr/Met) genotype was observed to elevate the risk of BC development in women with elevated BMI [OR: 1.66 (95% CI: 1.09–2.54); p = 0.019]. Similarly, the homozygous mutant (Met/Met) genotype was associated with the development of pre-menopausal BC [OR: 2.47 (95% CI: 1.05–5.80); p = 0.039].

Analysis of *RAD51* G135C variant and BC clinicopathological features revealed that the heterozygous (G/C) genotype reduced BC risk in women with elevated BMI (overweight and obese) [OR: 0.57 (95% CI: 0.36–0.91); p = 0.017]. On the other hand, the homozygous mutant (C/C) genotype was observed to confer an elevated risk of metastasis in women carrying the C/C genotype [OR: 2.41 (95% CI: 1.03–5.62); p = 0.042] (Table 4).

Concerning the *XRCC2* Arg188His variant and clinicopathological characteristics, we did not observe a significant association between various BC clinicopathological characteristics and Arg188His polymorphism in the breast cancer patients (S1 Table).

### MDR analysis

**Fig 1:**
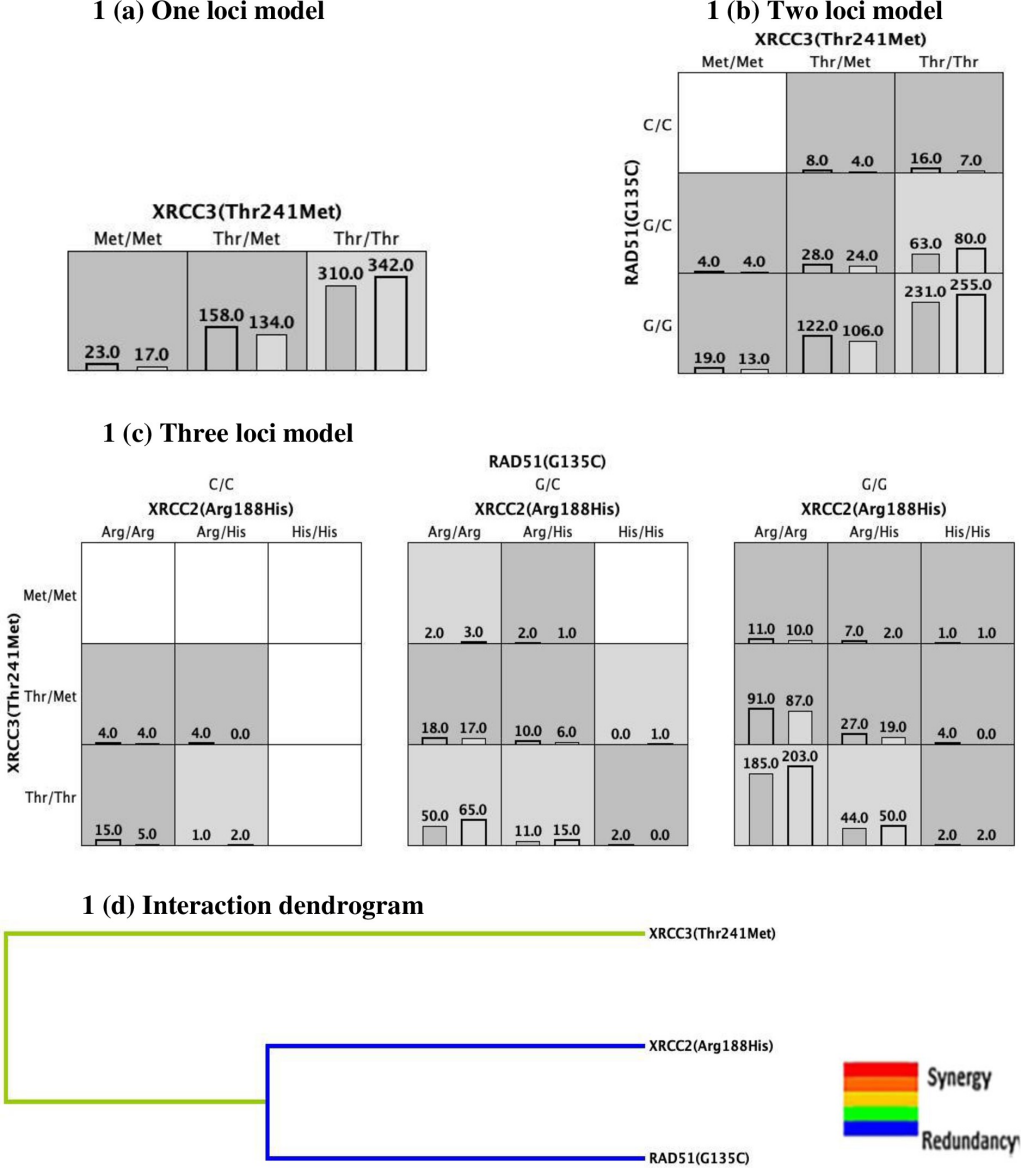
MDR analysis. Each cell depicts the number of BC cases on the left and the number of controls on the right. A high-risk genotype combination is given in dark grey cells, and a low-risk genotype combination is given in light grey cells. (a) Single-loci model representing cases and controls based on *XRCC3* (Thr241Met) variant, (b) two-loci model depicting cases and controls classified based on two SNPs (*XRCC3* -Thr241Met and *RAD51* –G135C), (c) three-loci model depicting cases and controls classified based on three SNPs, (d) interaction dendrogram revealed that the investigated HRR variants were found to have a redundant effect on BC development.

The combined effect of HRR gene variants (*XRCC2*-Arg188His, *XRCC3*-Thr241Met and *RAD51*-G135C) on BC risk was evaluated using MDR analysis. MDR evaluates the effect of SNP-SNP interaction on the risk of developing a multi-factorial disease such as breast cancer. MDR divides the data into a training dataset (9/10) and an independent testing dataset (1/10). A higher TBA value indicates that the observed interaction accurately predicts the case-control status. Moreover, a TBA score greater than 0.5 indicates that the interaction combination observed is not by chance, and a score of 1.00 highlights that the observed interaction combination is the best [27]. Furthermore, the model that has the highest TBA and CVC can be identified as the best interaction model. Fig 1 represents the various models predicted by MDR.

**Fig 2:**
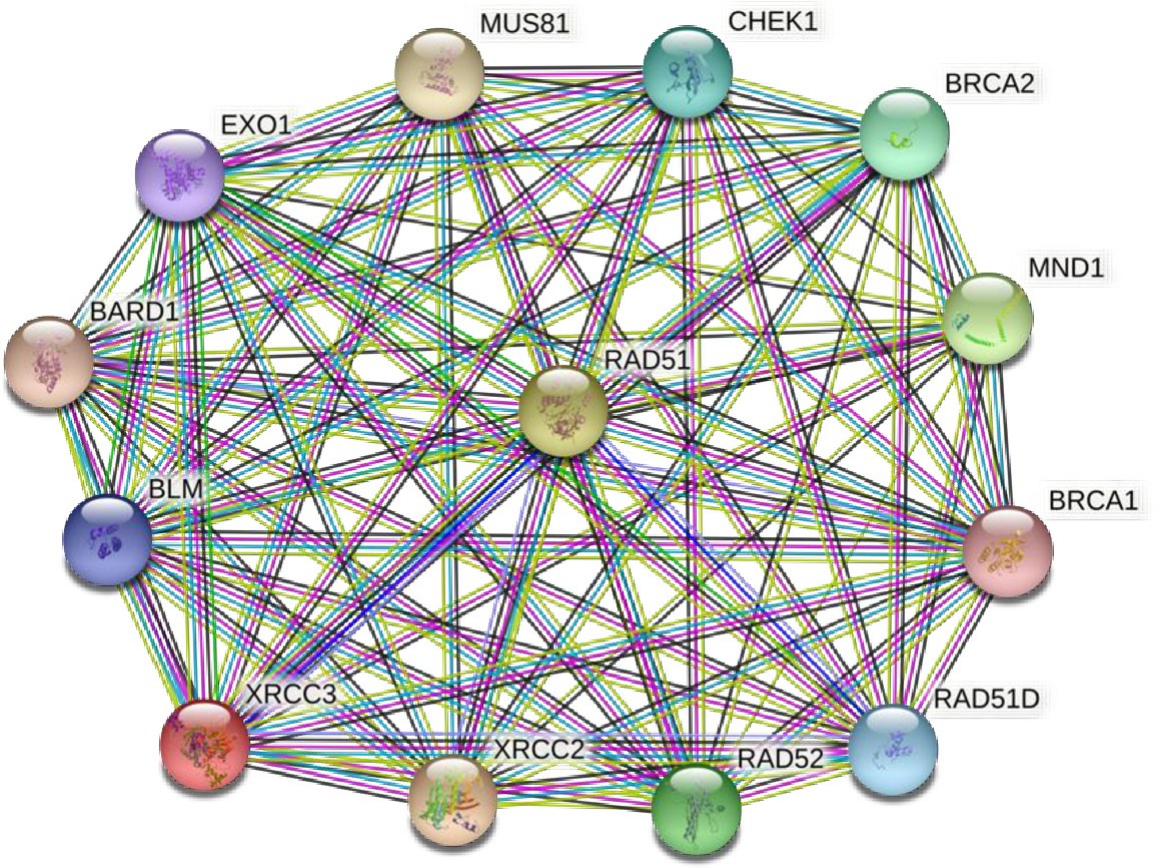
Protein-protein interaction. STRING software depicts the protein-protein interaction network of XRCC2, XRCC3 and RAD51. The first ten proteins that primarily interacts with XRCC2, XRCC3 and RAD51 is highlighted.

In the present study, MDR analysis revealed that among the different models, the interaction between *XRCC3*-Thr241Met and *RAD51*-G135C variants were observed to be the best interacting model under the two-loci model (TBA: 0.538; CVC: 10/10). Further investigation of the combinatorial impact of HRR variants on BC risk showed that the *XRCC2* - Arg/Arg, *XRCC3* -Thr/Thr, and *RAD51* - C/C genotype combination was elevated in BC cases compared to controls, thereby conferring elevated risk of BC [OR: 3.29 (95%CI: 1.17–9.23); p = 0.024] (Table 5). Fig 2 depicts the first ten protein partners that interact with XRCC2, XRCC3, and RAD51, which includes BRCA1, BRCA2, and RAD51D proteins. The STRING database collects and integrates all the publicly available protein-protein interaction sources and aids in the visualization of both direct (physical) and indirect (functional) interactions [28].

**Table 5:** Investigation of combinatorial impact of SNPs on BC risk.

Genotype combination (*XRCC2*, *XRCC3*, *RAD51*)	BC Cases (N)	Controls (N)	OR (95% CI)	*p*-value
Arg/Arg, Thr/Thr, G/G	185	203	Reference	
Arg/His, Thr/Met, G/G	27	19	1.56 (0.83–2.89)	0.160
Arg/His, Met/Met, G/G	7	2	3.84 (0.78–18.72)	0.096
Arg/Arg, Thr/Thr, G/C	50	65	0.84 (0.56–1.28)	0.428
Arg/Arg, Thr/Thr, C/C	15	5	**3.29 (1.17–9.23)**	**0.024**

## Discussion

Screening for certain commonly occurring polymorphisms has advanced our understanding of the crucial role played by genetics in BC predisposition. Moreover, various studies have reported that subtle variation in DNA repair capacity caused by the combination of low-penetrant genes and other influential factors such as environment modulates BC risk. Also, defects in DNA DSB repair has been identified as a common denominator for mammary carcinogenesis. Moreover, the *RAD51* gene family, including *XRCC2*, *XRCC3*, and *RAD51*, is highly polymorphic in nature [29]. Previous reports on the impact of HRR pathway gene variations from various ethnicities have yielded inconsistent results. Hence, the present study aims to clarify the role of genetic variants in crucial HRR genes (*XRCC2*, *XRCC3*, and *RAD51*) towards BC development.

In the present study, we found that the *XRCC2* Arg188His variant was not associated with BC predisposition in South Indian women. In line with our report, various studies observed a similar lack of association in Pakistani [30], Caucasian [26], Portuguese [31], African-American and white [32] women. Similarly, various meta-analysis studies investigating the role of the *XRCC2* Arg188His variant on BC development reported that the *XRCC2* Arg188His variant was not directly associated with BC predisposition [33–35]. Interestingly, a study by Silva *et al*. [31] highlighted that individuals who have never-breast fed and are heterozygous (Arg/His) for the *XRCC2* rs3218536 variant had reduced risk for BC.

Investigating the role of *XRCC3* Thr241Met variant and BC risk, we observed that the Met allele was associated with BC risk. However, in the present study, we found that the heterozygous (Thr/Met) and homozygous mutant (Met/Met) genotypes were not significantly associated with BC risk. A recent meta-analysis study based on 55 case-control studies on *XRCC3* Thr241Met variant and BC risk concluded that the *XRCC3* Thr241Met variant was associated with BC risk in Arabian and Asian populations [36]. Additionally, another meta-analysis concluded that the *XRCC3* Thr241Met variant was associated with a weakly elevated BC risk [37]. Similarly, another study on Thai [38] and South American [24] women reported that the 241Met carriers were at elevated BC risk. Furthermore, a study by Santos et al. [39] additionally observed that the *XRCC3* Thr241Met variant was slightly associated with an increased risk of BC in individuals with elevated chromosomal damage. However, reports from certain ethnicities have observed a lack of association between *XRCC3* Thr241Met variant and BC risk [25, 26, 40–43].

Interestingly, various studies reported that the *RAD51* G135C variant modified BC risk in *BRCA2* mutation carriers [44, 45]. Antoniou *et al*. [44] suggested that the *RAD51* G13C variant located in the 5’UTR region might also affect alternate splicing. Thus, the *RAD51* 135C allele might cause an overall decrease in RAD51 protein abundance. The present study investigated the role of *RAD51* G135C variant and BC risk, and we observed that the homozygous mutant (C/C) genotype was associated with an elevated risk of BC in South Indian women. A similar association was observed in a study conducted on mixed ethnicity (subjects were pooled from 19 studies including 13 countries) [44], Polish [40], and European [22] women. We also observed that the mutant CC genotype elevated the risk of metastasis in individuals with the homozygous mutant genotype. In line with our report, Weigmans et al. [46] speculated that breast tumors that overexpress *RAD51* might have an elevated chance of disease progression and metastasis. Additionally, they observed that *RAD51* promotes the expression of pro-metastatic genes and decreases the metastasis suppressor gene expression. Several meta-analyses highlighted the *RAD51* G135C variant could function as a potential candidate biomarker for various cancers, particularly breast cancer [47–49]. Sekhar *et al*. [50] observed that the homozygous mutant variant (C/C) elevated BC risk in an ethnic-specific manner. However, on the other hand, few studies suggested that *RAD51* tolerates very minimal dysfunctional sequence variation, and the *RAD51* G135C variant might not contribute towards BC susceptibility [23, 26, 51]. In contrast, another study in Polish women highlighted that the *RAD51* 135C allele reduced the risk of BC in *BRCA1* 5382insC mutation carriers [52]. Furthermore, the identification of *RAD51* foci in g*BRCA* mutant tumors was correlated with PARP inhibitor resistance [53, 54]. Overall, *RAD51* could be therapeutically targeted in the future using small molecule inhibitors [55] and could be used as a marker and target in neoadjuvant endocrine treatment [56].

Up to our knowledge, the present study is the first to report the impact of genetic polymorphisms in crucial HRR gene on BC development in South Indian women. Future studies that investigate the mechanistic role of HRR gene polymorphisms on BC predisposition, and studies that evaluate the prognostic potential of these SNPs might enhance our current understanding of the precise role played by these variants towards mammary carcinogenesis. Investigating common genetic variants that predispose BC development in developing nations such as India might aid primary health care providers in formulating schemes that could enable early diagnosis and lessen disease burden.

## Supporting information

S1 File Genotyping results of the study participants. (XLSX)

S1 Table Association between *XRCC2* Arg188His variant and BC clinicopathological characteristics. (DOCX)
