# Supplementary material for: Homologous recombination DNA repair gene RAD51, XRCC2 & XRCC3 polymorphisms and breast cancer risk in South Indian women
Source: PLoS One. 2022 Jan 21;17(1):e0259761. doi: 10.1371/journal.pone.0259761 (PMC8782413; doi:10.1371/journal.pone.0259761)
Supplement: S1 Table — (DOCX) [file pone.0259761.s002.docx]

| **Characteristics** | **Wild N (%)** | **Hetero N (%)** | **Mutant N (%)** | **Characteristics** | **Wild N (%)** | **Hetero N (%)** | **Mutant N (%)** |
| --- | --- | --- | --- | --- | --- | --- | --- |
| **Tumor Grade** |  | | | **Metastasis** |  | | |
| High (GII+GIII) | 316 (84.0%) | 86 (81.1%) | 8 (88.9%) | Positive | 92 (24.5%) | 27 (25.5%) | 1 (11.1%) |
| Low (GI) | 60 (16.0%) | 20 (18.9%) | 1 (11.1%) | Negative | 284 (75.5%) | 79 (74.5%) | 8 (88.9%) |
| OR (95%CI) | Reference | 0.82 (0.47-1.43) | 1.52 (0.19-12.37) | OR (95%CI) | Reference | 1.05 (0.64-1.73) | 0.39 (0.05-3.13) |
| p-value |  | 0.477 | 0.696 | p-value |  | 0.832 | 0.372 |
| **Tumor Stage** |  | | | **BMI** |  | | |
| Advanced (TIII+TIV) | 144 (38.3%) | 38 (35.8%) | 4 (44.4%) | Elevated BMI (≥ 25) | 253 (67.3%) | 65 (61.3%) | 6 (66.7%) |
| Early (TI+TII) | 232 (61.7%) | 68 (64.2%) | 5 (55.6%) | Normal BMI ( ≤24.9) | 123 (32.7%) | 41 (38.7%) | 3 (33.3%) |
| OR (95%CI) | Reference | 0.90 (0.57-1.41) | 1.29 (0.34-4.88) | OR (95%CI) | Reference | 0.77 (0.49-1.20) | 0.97 (0.24-3.95) |
| p-value |  | 0.650 | 0.709 | p-value |  | 0.253 | 0.969 |
| **ER Status** |  | | | **Menopausal status** |  | | |
| Negative | 161 (42.8%) | 48 (45.3%) | 4 (44.4%) | Premenopausal | 108 (28.7%) | 39 (36.8%) | 1 (11.1%) |
| Positive | 215 (57.2%) | 58 (54.7%) | 5 (55.6%) | Postmenopausal | 268 (71.3%) | 67 (63.2%) | 8 (88.9%) |
| OR (95%CI) | Reference | 1.10 (0.72-1.70) | 1.07 (0.28-4.04) | OR (95%CI) | Reference | 1.44 (0.92-2.27) | 0.31 (0.03-2.51) |
| p-value |  | 0.651 | 0.922 | p-value |  | 0.112 | 0.273 |
| **PR Status** |  | | | **Age at onset** |  | | |
| Negative | 210 (55.9%) | 62 (58.5%) | 7 (77.8%) | Young (≤40 years) | 50 (13.3%) | 22 (20.8%) | 0 (0%) |
| Positive | 166 (44.1%) | 44 (41.5%) | 2 (22.2%) | Old (>40 years) | 326 (86.7%) | 84 (79.2%) | 9 (100%) |
| OR (95%CI) | Reference | 1.11 (0.72-1.72) | 2.77 (0.57-13.49) | OR (95%CI) | Reference | 1.71 (0.98-2.98) | 0.34 (0.02-5.94) |
| p-value |  | 0.628 | 0.208 | p-value |  | 0.059 | 0.460 |
| **HER2 Status** |  | | | **Molecular subtype** |  | | |
| Positive | 156 (41.5%) | 46 (43.4%) | 2 (22.2%) | TNBC | 74 (19.7%) | 21 (19.8%) | 2 (22.2%) |
| Negative | 220 (58.5%) | 60 (56.6%) | 7 (77.8%) | Others | 302 (80.3%) | 85 (80.2%) | 7 (77.8%) |
| OR (95%CI) | Reference | 1.08 (0.69-1.67) | 0.40 (0.08-1.96) | OR (95%CI) | Reference | 1.01 (0.59-1.73) | 1.17 (0.24-5.73) |
| p-value |  | 0.725 | 0.261 | p-value |  | 0.976 | 0.850 |

S1 Table: *XRCC2* Arg188His variant and BC clinicopathological characteristics
